# A randomized trial comparing weight loss treatment delivered in large versus small groups

**DOI:** 10.1186/s12966-014-0123-y

**Published:** 2014-09-24

**Authors:** Gareth R Dutton, Lisa M Nackers, Pamela J Dubyak, Nicole C Rushing, Tuong-Vi T Huynh, Fei Tan, Stephen D Anton, Michael G Perri

**Affiliations:** Division of Preventive Medicine, University of Alabama at Birmingham, 1717 11th Avenue South, Medical Towers 615, Birmingham, AL 35205 USA; Department of Clinical and Health Psychology, University of Florida, Gainesville, FL USA; Department of Psychology, Florida State University, Tallahassee, FL USA; Department of Mathematical Sciences, Indiana University-Purdue University Indianapolis, Indianapolis, IN USA; Department of Aging and Geriatric Research, University of Florida, Gainesville, FL USA

**Keywords:** Weight loss, Group size, Lifestyle intervention, Randomized trial

## Abstract

**Background:**

Behavioral interventions for obesity are commonly delivered in groups, although the effect of group size on weight loss has not been empirically evaluated. This behavioral weight loss trial compared the 6- and 12-month weight changes associated with interventions delivered in a large group (LG) or small groups (SG).

**Methods:**

Obese adults (N = 66; mean age = 50 years; mean BMI = 36.5 kg/m^2^; 47% African American; 86% women) recruited from a health maintenance organization were randomly assigned to: 1) LG treatment (30 members/group), or 2) SG treatment (12 members/group). Conditions were comparable in frequency and duration of treatment, which included 24 weekly group sessions (months 1–6) followed by six monthly extended care contacts (months 7–12). A mixed effects model with unstructured covariance matrix was applied to analyze the primary outcome of weight change while accounting for baseline weight and dependence among participants’ measurements over time.

**Results:**

SG participants lost significantly more weight than LG participants at Month 6 (−6.5 vs. -3.2 kg; *p* = 0.03) and Month 12 (−7.0 vs. -1.7 kg; *p* < 0.002). SG participants reported better treatment engagement and self-monitoring adherence at Months 6 and 12, *p*s < 0.04, with adherence fully mediating the relationship between group size and weight loss.

**Conclusions:**

Receiving obesity treatment in smaller groups may promote greater weight loss and weight loss maintenance. This effect may be due to improved adherence facilitated by SG interactions. These novel findings suggest that the perceived efficiency of delivering behavioral weight loss treatment to LGs should be balanced against the potentially better outcomes achieved by a SG approach.

## Background

Behavioral interventions for obesity typically result in body weight reductions of 8-10% during the initial six months of treatment [1-3]. While treatment may be offered in group or individual settings, it is commonly delivered in groups of 8–15 participants [1,4,5]. Studies suggest that participants receiving group-based treatment lose significantly more weight than those receiving one-on-one treatment [6-8]. Group-based treatment has resulted in greater weight loss even among participants who expressed initial preference for individual treatment [7] and when treatment was delivered by telephone (i.e., group vs. individual calls) [6].

Potential reasons for the superior efficacy of group-based obesity treatment include the provision of additional social support, empathy, role-modeling of healthy choices, and a healthy dose of competition [4,9-11]. Rooted in social-cognitive theory [12], group-based lifestyle interventions utilize interaction among members to promote positive changes in self-efficacy for increased weight loss [13]. Through vicarious learning, verbal persuasion, and emotional and physiological activation, group members can impact each other and encourage behavior change [13].

While clinical experience and/or practical considerations (e.g., space constraints) have driven the traditional delivery of obesity treatment to groups of 8–15 individuals, research is lacking on whether this is more efficacious than alternative group sizes. A limited number of studies in the psychotherapy literature compared groups of differing size for the treatment of conditions such as anxiety, depression, and stress, but results have been mixed. While some studies found few differences between large and small groups [14], at least one demonstrated sustained improvements in anxiety symptoms and better session attendance with groups of 10–12 members versus larger groups of 20 members [15]. For interventions focused on changing health behaviors, one smoking cessation study found no association between group size and outcomes [16].

Participants within smaller groups may experience greater benefit regarding social support [17] and group cohesion [18]. Group-based obesity treatments capitalize upon positive group interactions to promote social learning, reinforcement, and support that ultimately lead to behavior change [4,9-13]. Given there are more opportunities for meaningful social interactions, smaller groups may promote higher quality, cohesive relationships among participants [17]. With the greater opportunity for social interaction and feedback, smaller groups could also promote increased self-efficacy and adherence (e.g., session attendance, completion of self-monitoring records) to treatment recommendations [13], which are strongly associated with weight loss success [19-23]. To our knowledge, however, no previous study has empirically tested the effects of group size on weight loss and related outcomes.

Examining the association between group size and treatment outcomes has important clinical and practical implications for weight loss treatment. Given the limited resources often available in applied clinical settings, treatment offered in larger groups may maximize available staff time and other organizational resources while promoting clinically significant weight loss. However, the delivery of group-based interventions to larger numbers of individuals has not been empirically evaluated. Therefore, it is important to compare the outcomes achieved with traditional, smaller groups and larger groups, as this decision about group sizes has direct implications for the implementation, dissemination, and sustainability of evidence-based weight loss treatments into “real world” settings.

This study examined the effect of group size on weight loss within a 12-month randomized behavioral weight loss intervention delivered in a managed care setting. Participants were randomized to receive treatment in either a “small” group (i.e., approximately 12 members per group, SG) typical of most group-based interventions [1,4,5,15] or a “large” group (i.e., approximately 30 members per group, LG). Given the theoretical rationale for delivering obesity treatment in a group format [12,13] along with previous results indicating the potential benefits of smaller groups [15,17,18], it was hypothesized that SG participants would demonstrate greater weight loss, better treatment attendance, better dietary self-monitoring adherence, and a more positive attitude toward the group than LG participants. Additionally, this study explored potential social (e.g., group climate) and behavioral (e.g., self-monitoring adherence) mediators of the association between group size and weight change.

## Methods

### Participants

Members of a health maintenance organization (HMO) located in northern Florida were recruited for participation. HMO members were men and women who either self-referred or received physician referral to the program. HMO members were made aware of the study through the HMO’s website, member newsletters, and during physician office visits. Members attended an initial information session, and interested members completed a brief screening assessment by telephone to determine eligibility.

Participants were ≥21 years-old, had a body mass index (BMI; kg/m^2^) between 30–45, and had current insurance coverage through the HMO. Individuals were excluded if they had lost >4.5 kg in the last six months, had a medical condition likely to affect participation in physical activity, planned to relocate from the coverage area in the next 12 months, were unable or unwilling to attend weekly group sessions, or were unwilling to accept random assignment to treatment groups.

**Figure 1:**
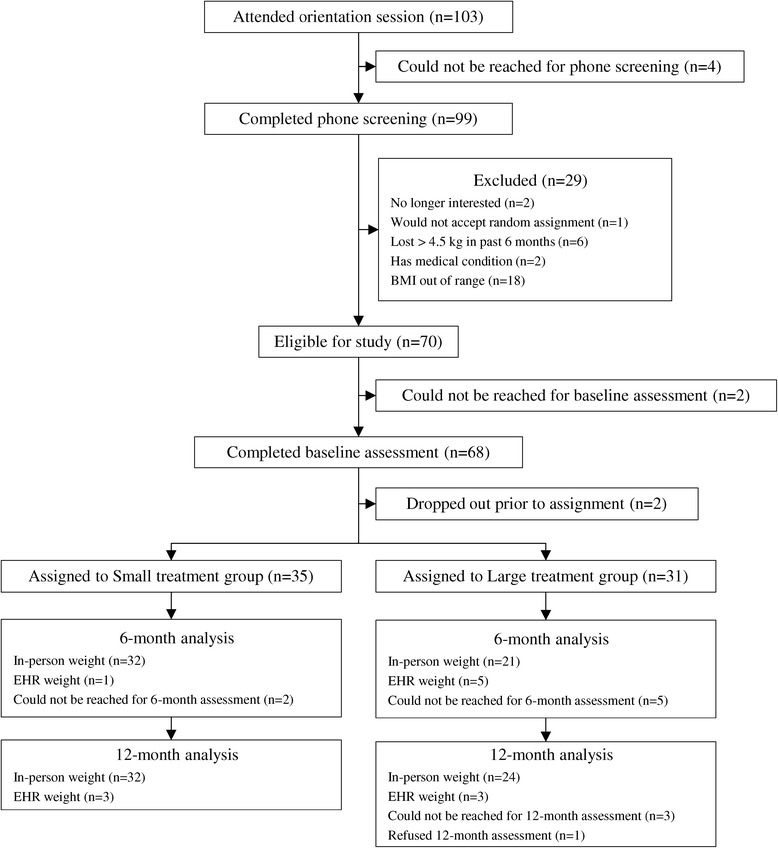
**Screening, randomization, and assessments of study participants.**

Among 103 individuals who attended the informational session, 99 completed a subsequent telephone screening and 70 were deemed eligible for participation. Of these, 68 individuals provided informed consent and completed a baseline assessment at the medical center; however, two individuals dropped out prior to treatment assignment. Thus, 66 participants were randomly assigned to the treatment conditions (Figure 1). Thirty-one individuals were assigned to one LG, and 35 individuals were assigned to one of three SGs (approximately 12 individuals/group).

Prior to recruitment, four meeting times for treatment groups were determined based on availability of clinic staff and space, and participants were asked to provide their availability for each of the four times during the screening process. After all participants provided availability, a computerized random number generator was used to determine which of the four meetings would be small or large groups. Participants were de-identified (represented by study ID and meeting time availability only) in order for staff to assign them to one of the four groups. Thus, the research team was masked to the identity of participants during treatment assignment (rather than being masked to condition itself) until all assignments had been made. Approval for this study was obtained from the IRBs of participating institutions.

### Measures

#### Demographics and medical history

At baseline, participants reported their age, sex, race, education level, marital status, and current tobacco and alcohol use. Participants also self-reported a history (yes/no) of the following medical conditions: high blood pressure, heart attack, chest pain, type 2 diabetes, gestational diabetes, pre-diabetes, arthritis, sleep apnea, high cholesterol, dizziness/fainting, asthma or chronic lung disease, and orthopedic problems. The total number of medical conditions was computed for each participant.

#### Weight and height

Weight and height were measured at each assessment in the medical clinic and used to calculate BMI. Weight was measured to the nearest 0.1 kg using a calibrated digital scale. Height was measured to the nearest 0.1 cm using a wall-mounted stadiometer. For participants who failed to complete the Month 6 and/or Month 12 assessment, recent clinic visit weights were accessed via electronic health records (EHR). EHR weights were imputed if they were recorded within an eight-week period (+/− four weeks) of the study assessment. EHR weights were used for six participants at Months 6 and 12.

#### Treatment attendance

Group leaders recorded participant attendance at group sessions. If a participant missed a group session, an individual make-up session was not provided, although the participant received session materials for review.

#### Self-monitoring adherence

Participants were instructed to self-monitor food intake using forms provided. Instructions included daily self-monitoring during Months 0–6 (i.e., up to 168 days of completed logs) and 3 days/week during Months 7–12 (i.e., up to 72 days of completed logs), which resulted in a total possible 240 days of completed logs. A completed log was defined as having at least two designated meals or eating episodes recorded within the day.

#### Treatment climate

Participants completed the Group Climate Questionnaire-Short Form (GCQ-S), a widely used measure of group process [24], at Months 6 and 12. The GCQ-S consists of 12 items rated on a six-point scale. The GCQ-S provides scores on three domains: Engagement, Conflict, and Avoidance [24]. For each domain, higher scores indicate higher perceived levels of that group process. The Engagement scale measures the extent to which the working group atmosphere is positive and cohesive. The Conflict scale reflects the amount of interpersonal anger and friction in the group. The Avoidance scale measures the extent of avoidance of responsibility for change by group members. The GCQ-S has demonstrated satisfactory reliability (Cronbach’s α ranging from 0.72-0.95) [25-27].

### Study design

#### Treatment description

The 12-month intervention was modeled after the lifestyle intervention of the Diabetes Prevention Program [28] and the treatment protocols developed and evaluated by Perri and colleagues [19]. Grounded in social-cognitive theory [12], these interventions seek to enhance individuals’ social support, personal motivators, and self-efficacy for behavior change [5]. Further, they have demonstrated efficacy for producing clinically meaningful weight reduction [19,29]. The format and content of sessions were the same for both conditions. Participants received pedometers, food scales, and measuring cups/spoons to facilitate self-monitoring and behavior change, and group leaders distributed self-monitoring logs at each session.

During Months 0–6, participants attended 24 weekly 90-minute group sessions. Prior to each session, participants were weighed privately. Each session included presentation, discussion, and practice of skills related to nutrition, exercise, and other self-management strategies. Participants also received training in self-monitoring, problem-solving, stimulus control, cognitive restructuring, and relapse prevention. Consistent with NHLBI guidelines [30], participants were encouraged to work towards a 10% reduction in body weight during the initial six-month period. To achieve this goal, participants were instructed to reduce caloric intake to 1,200 kcal/day (for participants weighing <250 pounds) or 1,500 kcal/day (for participants weighing ≥250 pounds). Participants were also encouraged to increase levels of moderate-intensity physical activity to 180 minutes/week.

Participants attended six monthly extended care sessions between Months 6–12. The purpose of extended-care was to maintain adherence, bolster motivation, and reinforce information previously discussed with a focus on the maintenance of healthy lifestyle behaviors. All study-related visits were held at one of the HMO outpatient clinics.

#### Intervention staff

All interventions were facilitated by a licensed clinical psychologist with expertise in delivery of weight management programs and/or doctoral students in clinical and counseling psychology. Each group included two co-facilitators. The intervention team met weekly to discuss all aspects of treatment delivery.

### Statistical analyses

Descriptive statistics, including sample mean and sample proportions, were used to summarize sample characteristics. T-tests were used to compare population means, and *χ*^2^ tests or Fisher’s exact tests were used to assess association between categorical variables. To analyze the main outcome of weight change over time while accounting for the dependence among measurements of the same participant, we applied a mixed effects model with unstructured covariance matrix and adjusted for baseline body weight. Without imputation, this method allowed for participants with missing measurements at either follow-up (n = 7 at Month 6; n = 4 at Month 12) to be retained in analyses. Comparison of attendance, adherence, and group climate scores (i.e., Engagement, Conflict, and Avoidance scales of the GCQ-S) for the SG and LG conditions were examined using t-tests at Months 6 and 12. Cumulative attendance was analyzed at Month 12.

Evaluating measures of group climate (i.e., Engagement, Conflict, and Avoidance), attendance, and adherence as potential mediators of the association between group assignment and weight change were proposed. To explore this, additional regression analyses were conducted based on the mediation approach initially described by Baron and Kenny [31] and recently updated by Cerin and MacKinnon [32], as well as the multiple mediator modelling method detailed by Preacher and Hayes [33]. In this approach, the change between overall treatment effect and the residual treatment effect of group size on weight change, after simultaneously accounting for the impact of the specified mediator variables, were examined using the product-of-coefficient estimate method. First, a test of the action theory was performed examining the association between treatment and the proposed mediators. For constructs demonstrating a significant association with group size, a test of the conceptual theory further assessed the relationship between the mediators and the outcome (i.e., weight change), while adjusting for the effect of treatment in the model. In these analyses, baseline weight was included as a confounder variable. For each mediator, the point estimator of mediation effect was calculated as the product of the action theory test and the conceptual theory test regression coefficients. Due to small sample size and subsequent skewed distributions of path effects, we obtained bias-corrected bootstrap confidence intervals based on 10,000 bootstrap samples [32,34-36]. Also consistent with Cerin and MacKinnon [32], multiple-mediator models were employed, and mediation effect estimates were reported in the units of the outcome variable (kg). Statistical analyses were conducted using SAS/STAT® software, Version 9.3 of the SAS System for Windows, and R statistical software [37].

## Results

### Participant characteristics

Participant characteristics are summarized in Table 1. The majority of participants were women (86%), and nearly half were African American (47%). There were no significant differences between the LG and SG conditions for any of the baseline characteristics assessed, *p*s > 0.05.

**Table 1:** **Baseline characteristics of the total sample and each treatment condition**

	**Overall sample**	**LG**	**SG**	
	**(N = 66)**	**(n = 31)**	**(n = 35)**	
	**M (SD)**	**M (SD)**	**M (SD)**	***p*** **-value**
Age	50.2 (10.7)	48.1 (11.8)	51.9 (9.5)	0.151
Weight (kg)	102.3 (20.0)	104.4 (24.2)	100.5 (15.4)	0.441
BMI (kg/m^2^)	36.5 (5.7)	36.9 (6.3)	36.1 (5.2)	0.586
# of medical conditions	2.2 (1.8)	2.1 (1.6)	2.4 (2.0)	0.544
	%	%	%	
Female	86.4	83.9	88.6	0.724
African American	47.0	41.9	51.4	0.441
Education, graduated college	51.5	41.9	60.0	0.143
Married	59.1	58.1	60.0	0.873
Current tobacco use	4.6	6.5	2.9	0.597
Current alcohol use	47.0	51.6	42.9	0.477

### Effect of group size on weight loss

**Figure 2:**
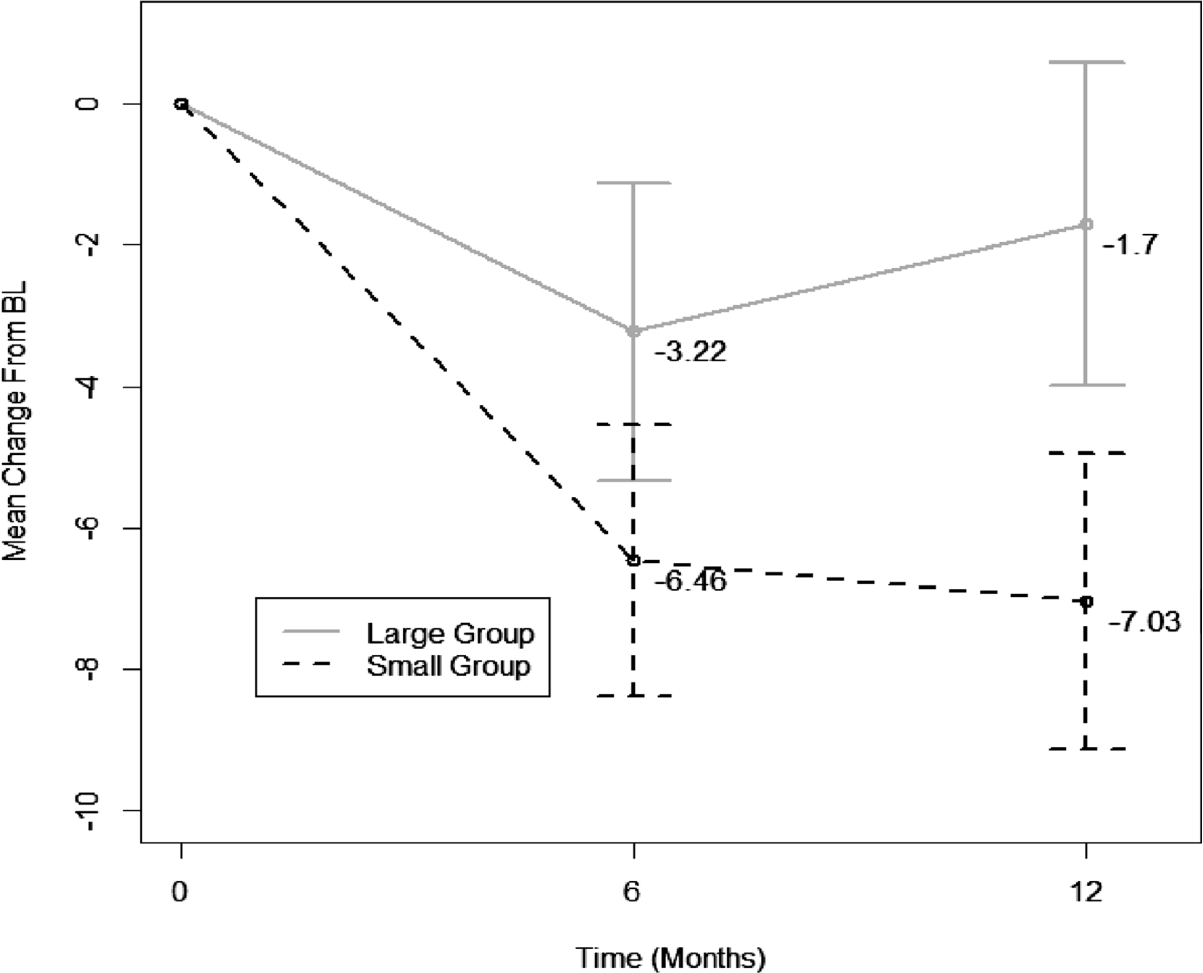
**Mean weight change over time by group.**

Participants in the LG condition lost an average of 3.2 kg from Month 0–6, *p* = 0.004. However, participants in the SG condition demonstrated an additional 3.2 kg of weight loss (mean loss = −6.5 kg), representing a significant difference between the LG and SG conditions at Month 6, *p* = 0.03. Participants in the LG condition experienced weight regain between Months 6–12, such that their mean weight at Month 12 was not significantly different from baseline (mean loss from baseline = −1.7 kg), *p* = 0.15. In contrast, SG participants continued to lose a small amount of weight between Month 6–12, and their Month 12 weights remained significantly lower than baseline (mean loss from baseline = −7.0 kg) and LG participants’ Month 12 weights (additional mean loss = −5.3 kg), *p* = 0.001 (see Table 2 and Figure 2).

**Table 2:** Analysis of weight change (kg) at Months 6 and 12*

**Independent variable**	**Estimate**	**Std Err**	**p-value**
LG mean baseline weight, kg (intercept)	104.39	3.60	<.0001
Difference in mean baseline weight (SG – LG)	−3.93	4.94	0.4286
Month 6 mean weight loss in LG	−3.22	1.08	0.0039
Month 6 additional mean weight loss in SG	−3.23	1.46	0.0301
Month 12 mean weight loss in LG	−1.70	1.17	0.1504
Month 12 additional mean weight loss in SG	−5.33	1.58	0.0013

*Results are based on mixed effects model with unstructured covariance matrix.

When examining the proportion of participants in each condition who achieved ≥ 5% weight loss, chi square analyses indicated that 57.6% of SG participants met this criterion at Month 6 compared with 42.3% of LG participants, which was a non-significant difference, *p* = 0.244. At Month 12, however, significantly more SG participants (51.4%) achieved a ≥ 5% weight loss compared to LG participants (25.9%), *p* = 0.042.

### Effect of group size on session attendance

Participants assigned to the SG condition attended approximately three more of the 24 weekly sessions than LG participants during the first six months of the program, although this was a non-significant difference, *p* = 0.095 (see Table 3). This difference in attendance rates remained when the six monthly extended care sessions offered between Months 6–12 were included, with SG and LG attendance rates of 18.6 and 14.7 (of 30 sessions), respectively, *p* = 0.089.

**Table 3:** **Comparison of SG and LG conditions on treatment attendance, adherence, and group climate**

	**SG**	**LG**	***p*** **-value**
Session attendance			
Baseline – month 6^a^	15.8 (7.0)	12.8 (7.4)	0.0947
Baseline – month 12^b^	18.6 (8.8)	14.7 (9.2)	0.0892
Self-monitoring adherence			
Baseline – month 6^c^	98.5 (47.3)	59.1 (56.8)	0.0042
Baseline – month 12^d^	113.0 (56.3)	68.2 (69.2)	0.0068
Group climate scores^e^			
Engaged (baseline – month 6)	4.3 (0.9)	3.7 (0.9)	0.0371
Engaged (baseline – month 12)	4.2 (1.1)	3.2 (0.8)	0.0004
Conflict (baseline – month 6)	0.2 (0.3)	0.3 (0.4)	0.2164
Conflict (baseline – month 12)	0.2 (0.4)	0.4 (0.5)	0.0784
Avoidance (baseline – month 6)	2.7 (1.1)	2.4 (1.1)	0.3506
Avoidance (baseline – month 12)	2.6 (1.2)	2.7 (1.0)	0.7696

^a^ of 24 sessions, ^b^ of 30 sessions, ^c^ of 168 days, ^d^ of 240 days, ^e^ score range = 0-6.

### Effect of group size on self-monitoring adherence

SG participants completed significantly more days of dietary self-monitoring than LG participants (mean difference = 39.4 days) during the first six months of treatment, *p* = 0.004 (Table 3). SG participants also completed more days of self-monitoring for the full 12 months of the program (mean difference = 44.8 days), *p* = 0.007.

### Effect of group size on treatment climate

Based on scores from the Group Climate Questionnaire (i.e., Engagement, Conflict, and Avoidance), results indicated that SG participants reported experiencing significantly better engagement in the treatment process than LG participants, and this difference was observed for the engagement scores at Months 6 and 12, *p*s < 0.04. The two conditions did not differ on perception of the group’s ability to effectively cope with conflict or avoid problems during the course of treatment (Table 3).

### Potential mediators of the association between group size and weight loss

**Figure 3:**
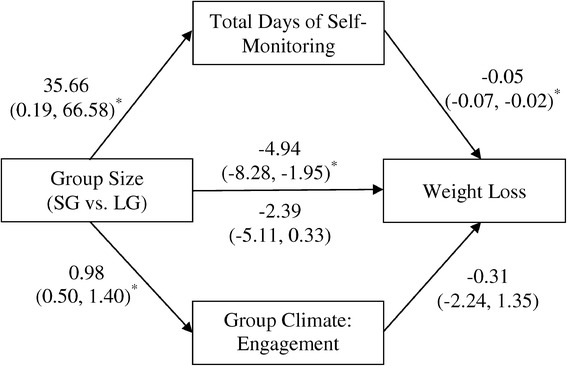
**Self-monitoring adherence and engagement as mediators of the association between group size and weight change.**

Additional analyses were conducted to examine whether treatment engagement and/or adherence mediated the relationship between treatment condition and weight loss. Results of this multiple mediator analysis (Figure 3) indicated that, compared to participants in the LG condition, those in the SG condition completed self-monitoring on 35.66 more days, 95% CI (0.19, 66.58), and there was a −0.05 kg weight loss, 95% CI (−0.07, −0.02), per each additional day of self-monitoring. Thus, the product of mediation path effects specifically associated with adherence (i.e., group size-to-adherence and adherence-to-weight loss) was −1.77 kg, 95% CI (−4.13, −0.14), indicating a significant additional 1.77 kg weight loss in SG attributable to this variable. Mediation analysis also indicated that the average engagement score of SG participants was 0.98 points, 95% CI (0.50, 1.40), higher than that of LG participants, although the weight change associated with each additional point increase in engagement score was not significant, −0.31 kg, 95% CI (−2.24, 1.35). Hence, the indirect effect of group size on weight loss through engagement was not significant at −0.31 kg, 95% CI (−2.56, 1.27). The total indirect effect of group size on weight change through the two mediators was −2.09 kg, 95% CI (−4.76, −0.09), indicating the treatment effect was significantly reduced after accounting for these variables. In addition, simultaneous inclusion of the proposed mediators of engagement and adherence reduced the magnitude of the association between SG assignment and weight loss from −4.94 kg, 95% CI (−8.28, −1.95) to a non-significant value of −2.39 kg, 95% CI (−5.11, 0.33). In summary, self-monitoring adherence fully mediated the association between group size and weight loss, while treatment engagement was not a significant mediator when also accounting for adherence. Because group size was not significantly associated with session attendance or the other subscale scores from the group climate measure, these variables were not included in mediation analyses.

## Discussion

The present study examined whether delivery of a 12-month behavioral weight loss intervention in either “small” or “large” group formats within a managed care setting impacted weight loss outcomes. The primary finding demonstrates that participants assigned to the SG condition lost significantly more weight at both 6 and 12 months of the intervention when compared to participants within the LG. Participants in both groups achieved significant weight losses at Month 6 (−6.4 kg for SG versus −3.2 kg for LG). Participants in the LG, however, experienced weight regain from Months 6–12, resulting in a final weight that did not significantly differ from baseline. Conversely, participants in the SG demonstrated slight additional weight loss from Months 6–12, such that by Month 12 they achieved a significant average loss of −7.03 kg, which is comparable to weight losses seen in other trials [29,38]. In addition, 51% of SG participants attained a 5% weight loss compared to only 26% in the LG at Month 12. A 5% body weight loss has clinical significance, resulting in reduced risk factors for diabetes and heart disease [29,38]. While group-based interventions have demonstrated superiority when compared to individually-tailored delivery [6-8], this study further suggests that delivery to smaller groups of approximately 12 participants, versus larger groups of approximately 30 participants, results in more successful weight loss.

Both session attendance and self-monitoring adherence are strongly associated with overall weight loss success in lifestyle interventions for obesity [19-23]. SG participants attended nearly four more sessions on average than LG members, although this was a non-significant difference. Additionally, adherence to self-monitoring differed significantly between conditions, with higher rates of adherence observed among SG participants. This difference in self-monitoring adherence mediated the association between group size and weight loss, such that the residual direct effects of group size were no longer significant after accounting for adherence. Smaller groups, as compared to larger groups, may provide more opportunities for interventionists and participants to reinforce the accuracy and completion of self-monitoring records, problem-solve barriers to maintaining records, and set individualized goals for improving adherence. Therefore, increased accountability in smaller groups may promote greater adherence and contribute to weight loss success.

Within the psychotherapy literature, smaller group size is associated with increased social support [17] and group cohesion [18]. Larger groups may limit potential resources available and may cause individual members difficulties in making connections, leading to feelings of isolation or alienation [17]. In the present study, SG participants endorsed a more positive and cohesive group atmosphere than LG members, suggesting that smaller groups allow for greater allocation of resources and improved opportunities for supportive interactions that foster an environment encouraging of weight loss. Treatment engagement did not, however, mediate the association between group size and weight loss after accounting for self-monitoring adherence. While it is possible that the included measure did not fully capture the most salient features of engagement, the more robust influence of self-monitoring adherence may have also contributed to these findings. According to social-cognitive theory [12], the cohesive bonds and meaningful interactions experienced among SG participants may have promoted increases in self-efficacy for weight loss behaviors, such as self-monitoring [13]. Indeed, members who feel connected within their group have demonstrated greater responses to group treatment, such as an increased willingness to disclose during discussion [39], higher goal attainment [40], and better attendance rates [41,42].

This study did have limitations. First, group members were recruited solely within HMOs, with the sample being comprised mainly of females (86%). While the sample was racially and educationally diverse, generalizability remains limited to other clinical settings. Second, the sample size was modest and may have been inadequately powered to detect some group differences. Third, the study timeline lasted one year. While participants in the SG continued to lose a small amount of weight from Months 6–12, it remains unclear whether this trend would continue in the long-term. Fourth, the LG condition included only one treatment group, while the SG condition included three groups that were combined for analyses. Thus, there was a potential cluster effect not fully accounted for by the analyses, as the inclusion of a single LG prohibited estimating random effects attributable to cluster (i.e., random cluster effect could not be separated from the treatment effect for the LG). Fifth, this trial only compared groups of approximately 12 versus 30 members, so the relative effects of moderate-sized groups (e.g., 15–30 participants) is unknown. Finally, while self-monitoring adherence fully mediated the association between group size and weight loss, there may be other process variables not assessed that additionally account for the weight change differences observed between the LG and SG conditions.

While this study provides novel information on the treatment effects of LG and SG conditions, cost-effectiveness of the two approaches was not evaluated. Even though SG participants achieved greater weight loss, it is important to consider whether LG treatment delivery remains a viable option from a cost-efficiency perspective. By formally measuring and calculating program operation costs (e.g., interventionist and administrator time, meeting space fees, etc.), future research could determine costs per kg of weight lost, or a similar metric of cost-effectiveness, to select the appropriate group size that maximizes weight loss at the lowest costs.

The present study also had a number of strengths. The trial utilized a randomized design, and retention was excellent with approximately 94% of participants contributing weight data at Month 12. In addition, all participants received a structured lifestyle intervention modeled after evidence-based protocols [19,28], which incorporated state-of-the-art behavioral principles to promote weight loss and maintenance. Given the nature of the HMO setting, access to electronic health records provided objective weight data for participants who did not attend follow-up study assessment visits.

In addition to these methodological strengths, this study offers important clinical implications for the delivery of behavioral weight loss treatment. There may be a perceived benefit to delivering evidence-based weight loss treatment to larger numbers of participants in clinical settings to efficiently use the limited resources available. However, this study suggests that clinicians and administrators may need to balance this potential advantage with the improved weight loss outcomes achieved by delivering treatment in smaller groups. Recently, researchers have investigated alternate, more cost-effective methods of weight loss intervention delivery, including group phone calls [6] and group internet-based programs [43]. Utilizing these alternative methods may retain group cohesiveness while incorporating more cost-effective modes of delivery and reducing burden on staff and organizational resources. Future studies should continue to assess whether alternative methods of delivery prove superior for weight loss, cost-effectiveness, and dissemination when delivered to traditional groups of 8–15 participants.

## Conclusions

To our knowledge, the present study represents the first empirical test of the effects of group size on weight loss outcomes. The findings suggest that providing lifestyle intervention for obesity in traditional groups of approximately 12 participants is superior to delivery in larger groups of 30 or more participants. Participants in the smaller groups achieved larger weight losses and were more likely to attain a clinically significant weight loss of ≥ 5%. A smaller group size also resulted in higher perceived group engagement, suggesting that smaller group settings provide more positive, cohesive, and collaborative atmospheres. Small groups also facilitated greater adherence to behavioral weight management strategies (i.e., self-monitoring), and this behavior mediated the relationship between group size and treatment outcomes. Future studies should continue to investigate the utilization of smaller group sizes with alternative modes of delivery (e.g., telephone, internet) to improve cost effectiveness within managed care and other applied clinical settings.

## Abbreviations

LG: Large group
SG: Small group
HMO: Health maintenance organization
